# Full-length MAVS, a mitochondrial antiviral-signaling protein, inhibits hepatitis E virus replication, requiring JAK-STAT signaling

**DOI:** 10.1007/s00705-022-05415-9

**Published:** 2022-03-24

**Authors:** Changbo Qu, Yang Li, Yunlong Li, Yihang Pan

**Affiliations:** 1 Tomas Lindahl Nobel Laureate Laboratory, Precision Medicine Research Center, The Seventh Affiliated Hospital of Sun Yat-sen University, Shenzhen, 518107 China; 2 Department of Biochemistry, Radboud Institute for Molecular Life Sciences, Radboud University Medical Center, Nijmegen, The Netherlands; 3 Radboud Center for Mitochondrial Medicine, Radboud University Medical Center, Nijmegen, The Netherlands; 4 Department of Gastroenterology and Hepatology, Erasmus MC-University Medical Center, 3015CN Rotterdam, The Netherlands

## Abstract

Hepatitis E virus (HEV) infection is the leading cause of acute hepatitis worldwide. The mitochondrial antiviral signaling protein (MAVS)-mediated interferon (IFN) response plays a pivotal role in hepatic antiviral immunity. However, little is known about the effect of overexpression of MAVS on HEV infection. Full-length MAVS (FL-MAVS) is the main form of MAVS that increases the production of IFNs. Here, we studied the effect of FL-MAVS on HEV infection. We found that overexpression of FL-MAVS profoundly inhibited HEV replication. Furthermore, we showed that the anti-HEV effect of FL-MAVS is largely dependent on JAK-STAT signaling activation.

**Supplementary Information:**

The online version contains supplementary material available at 10.1007/s00705-022-05415-9.

Hepatitis E virus (HEV) is a nonenveloped single-stranded RNA virus. Although only one serotype has been identified, various genotypes have been reported to infect humans [1]. Acute infection with HEV genotype 1 in pregnant women has been found to cause severe liver inflammation with high mortality [2]. Organ transplant recipients infected with HEV genotype 3 are at high risk of developing chronic hepatitis E with rapid progression to cirrhosis [3]. Mitochondria are cellular organelles that participate in various metabolic activities as well as the regulation of innate immunity [4]. The mitochondrial antiviral signaling protein (MAVS) is an adaptor protein that is primarily localized in the outer membranes of mitochondria and, to a lesser extent, in peroxisomes [5, 6]. Upon RNA virus infection, the viral genome is released into the cytoplasm to initiate viral protein biosynthesis. The released viral genome acts as a pathogen-associated molecular pattern (PAMP), which is recognized by proteins of the RIG-I-like receptor (RLR) family: RIG-I and MDA5 [7]. The caspase activation and recruitment domains (CARDs) of RIG-I and MDA5 bind to a CARD in the MAVS, resulting in MAVS aggregation, which then triggers the production and secretion of interferons (IFNs) [8]. The secreted IFNs then bind to their cell-surface receptors, resulting in the activation of the Janus kinase/signal transducer and activator of transcription (JAK-STAT) pathway and the subsequent induction of transcription of hundreds of interferon-stimulated genes (ISGs), which are usually considered to be the ultimate antiviral effectors [9]. Acting as a key adaptor for IFN signaling, MAVS shows specific interactions with different hepatitis viruses. For example, hepatitis C virus (HCV) NS3/4A protease blocks the interferon (IFN) response by cleaving the MAVS protein [10]. In parallel, a product of hepatitis A virus (HAV) polyprotein processing, 3ABC, targets MAVS for proteolysis [11]. Instead of directly provoking MAVS proteolysis or MAVS cleavage, HEV has been reported to induce MAVS polymerization, rendering the infected cells more resistant to exogenous IFNs [12]. The HEV-induced MAVS polymer represents a remarkable example of virus evolution, although the role of ectopic overexpression of MAVS in HEV infection remains unclear. Full-length MAVS (FL-MAVS) is the main form of MAVS that increases the production of IFNs [13]. In this study, we investigated the effect of FL-MAVS on HEV infection. We found that FL-MAVS overexpression significantly inhibited HEV replication. Mechanistically, we show that the anti-HEV effect of FL-MAVS is largely dependent on the activation of the JAK-STAT signaling pathway.

The effect of FL-MAVS on HEV was first tested in Huh7.5-cell-based HEV infectious (Huh7.5-p6) and replicon (Huh7.5-p6-Luc) models. We found that the FL-MAVS overexpression exerted a potent inhibitory effect on HEV in Huh7.5-p6 cells (Fig. 1A and B). Similarly, in Huh7.5-p6-Luc cells, an increased level of FL-MAVS also inhibited HEV replication after 48 h of treatment (Fig. 1C). PLC/PRF/5 (PLC) cells have been used to establish another infectious model of HEV genotype 3 (PLC-p6) [14]. In line with the results in Huh7.5-p6 cells, overexpression of FL-MAVS also significantly inhibited HEV replication in PLC-p6 cells (Fig. 1D). Next, the antiviral effect of FL-MAVS on HEV genotype 1 was assessed. We found that FL-MAVS overexpression significantly inhibited HEV genotype 1 replication, suggesting a broad activity of FL-MAVS against different HEV genotypes (Fig. 1E). As described previously, RIG-I, MDA5, and MAVS are key players in the IFN-mediated activation of JAK-STAT signaling, which plays a key antiviral role by driving the transcription of ISGs [8, 9, 15]. Our experimental studies have demonstrated that overexpression of RIG-I or MDA5 significantly inhibits HEV replication by stimulating the expression of a large variety of ISGs [9, 15]. In this study, we found that overexpression of FL-MAVS also promoted an antiviral state by robustly increasing the expression of ISGs in the HEV models (Fig. 2A and Supplementary Fig. S1A). Classically, upregulation of ISGs occurs following the IFN-induced activation of JAK-STAT signaling, which leads to the nuclear translocation and binding of the ISG factor 3 (ISGF3) complex to specific promoter elements in IFN-stimulated response elements (ISREs) [16]. To test the activation of this signaling, we employed an ISRE reporter system in which expression of a luciferase reporter gene was driven by multiple ISREs. The activity of JAK-STAT signaling can thus be monitored by measuring the ISRE luciferase activity (ISRE-RLU). Consistent with the upregulation of ISGs, FL-MAVS overexpression led to a clear increase in ISRE luciferase activity after 48 h of treatment (Supplementary Fig. S1B), suggesting that activation of JAK-STAT signaling had occurred. The JAK-STAT signaling activation requires binding of IFNs to their receptors; however, our previous studies showed that the increased levels of ISGs induced by RIG-I or MDA5 are not associated with functional IFN production during HEV infection [9, 15]. We next tested whether IFNs are produced during FL-MAVS overexpression. Interestingly, overexpression of FL-MAVS was found to increase IFN RNA in the Huh7.5-p6 model (IFNβ and IFNλ1) and the PLC-p6 model (IFNα, IFNλ1, and IFNλ2), as determined using a qRT-PCR assay (Fig. 2B and Supplementary Figure S1C). These results prompted us to further assess whether functional IFNs are secreted. To this end, conditioned medium (supernatant) from the FL-MAVS-transduced HEV models (Huh7.5-p6 and PLC-p6) was collected, and two IFN-sensitive assays (an IFN functional assay and an HCV replicon-based bioassay) were performed [9, 15]. Consistent with the upregulation of IFN RNA, functional IFN secretion was detected in the HEV models overexpressing FL-MAVS, as exemplified by the observation that the conditioned medium obtained from these models significantly increased ISRE activation (Fig. 2C and Supplementary Fig. S1D) and decreased HCV-related luciferase activity (Fig. 2D and Supplementary Fig. S1E). Of note, treatment of the HEV replicon model with the conditioned medium resulted in a minor decrease in HEV-related luciferase activity, suggesting a potential role of IFNs against HEV during FL-MAVS overexpression (Supplementary Fig. S1F). To further define the role of FL-MAVS-induced autocrine IFNs during HEV replication, two IFN binding inhibitors were used. B18R (which blocks IFNα/β receptor binding) and 136R (which preferentially blocks IFNλ receptor binding) are poxvirus-derived soluble IFN antagonists that have been used to antagonize IFN binding in Huh7 cells [17]. However, treatment with B18R or 136R did not significantly reverse the anti-HEV effect of FL-MAVS, as determined by qRT-PCR assay (Fig. 2E and F).

**Fig. 1:**
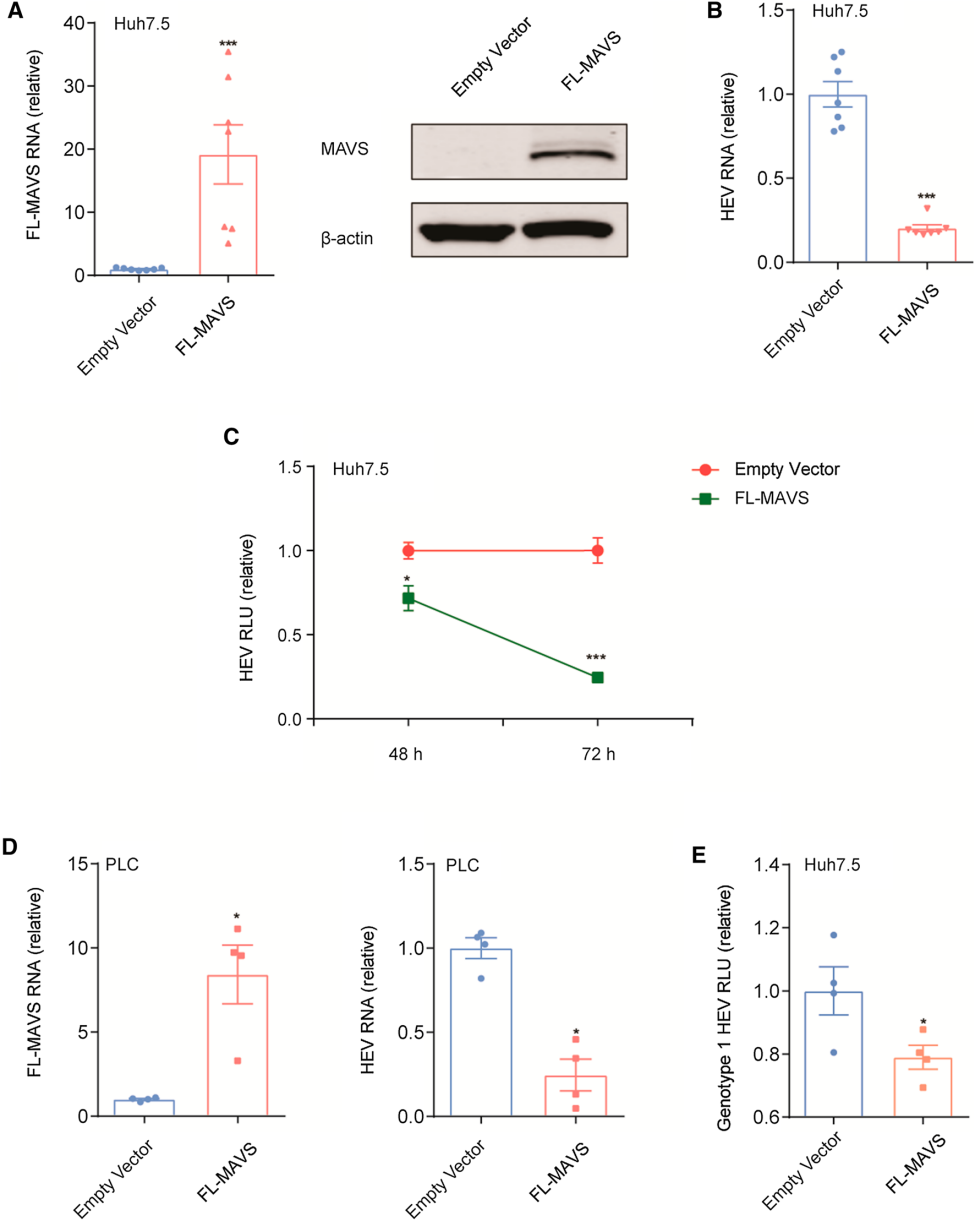
FL-MAVS inhibits HEV replication. (A) Quantitative RT-PCR analysis of FL-MAVS RNA (n = 7) and immunoblot analysis of the FL-MAVS protein level in Huh7.5-p6 cells transduced with FL-MAVS or empty vector for 48 h. MAVS was detected using anti-FLAG antibody. (B) Quantitative RT-PCR analysis of HEV RNA (n = 7) in Huh7.5-p6 cells transduced with FL-MAVS or empty vector for 48 h. (C) Analysis of HEV-related luciferase in Huh7.5-p6-Luc cells transduced with FL-MAVS or empty vector for the indicated time periods (n = 7). (D) Quantitative RT-PCR analysis of FL-MAVS RNA (n = 4) and HEV RNA (n = 4) in PLC-p6 cells transduced with FL-MAVS or empty vector for 48 h. (E) Analysis of HEV-related luciferase in a genotype 1 HEV cell model transduced with FL-MAVS or empty vector (n = 4) for 48 h. Data are the mean ± SEM (*, *P* < 0.05; **, *P* < 0.01; ***, *P* < 0.001).

**Fig. 2:**
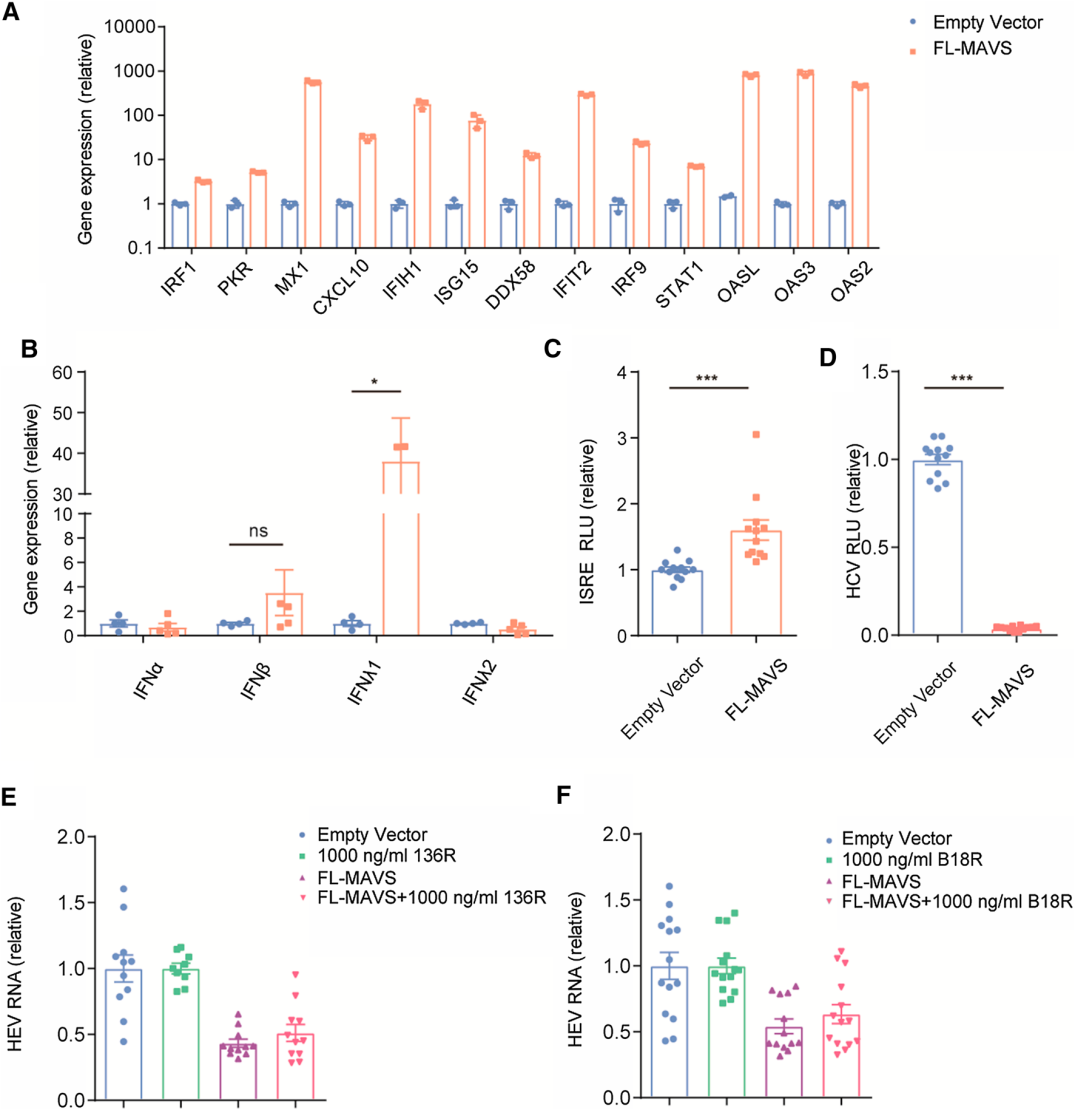
Overexpression of FL-MAVS induces IFN secretion. (A) Quantitative RT-PCR analysis of ISGs RNA in Huh7.5-p6 cells transduced with FL-MAVS or empty vector (n = 3) for 48 h. (B) Quantitative RT-PCR analysis of IFN RNA in Huh7.5-p6 cells transduced with FL-MAVS or empty vector (n = 4-5) for 48 h. Huh7.5-p6 cells were transduced with FL-MAVS or empty vector for 72 h. After that, the cells were washed five times with PBS, and the cell culture medium was refreshed. After another 72 h, the supernatant was collected as a conditioned medium. (C and D) Analysis of firefly luciferase activity in Huh7-ISRE-Luc cells (n = 12) (C) or in an HCV replicon model (n = 12) (D) treated with Huh7.5-p6-derived conditioned medium for 72 h. (E and F) The Huh7.5-p6 cell model was transduced with FL-MAVS or empty vector or treated with 1000 ng/ml 136R (n = 9-11) (E), 1000 ng/ml B18R (n = 13-14) (F), or 0.1% DMSO control for 48 h. HEV RNA was measured by quantitative RT-PCR. Data in the FL-MAVS group are presented relative to the empty vector group (set as 1). Data in the combination group of FL-MAVS with 136R or B18R are presented relative to the 136R- or B18R-alone-treated group (set as 1). The data are the mean ± SEM (*, *P* < 0.05; **, *P* <0.01; ***, *P* < 0.001).

To further explore the role of activated JAK-STAT signaling during FL-MAVS overexpression, a JAK inhibitor was used [15, 18]. We found that adding 10 μM of JAK inhibitor robustly reversed the anti-HEV effects of FL-MAVS in Huh7.5-p6 and Huh7.5-p6-Luc cells (Fig. 3A and B), suggesting that the JAK-STAT signaling pathway is largely required for FL-MAVS-mediated inhibition of HEV. The phosphorylation of STAT1 at the position 701 (P-STAT1) is an indispensable marker of JAK-STAT pathway activation [9]. Western blot analysis showed that FL-MAVS overexpression increased the level of P-STAT1 in Huh7.5-p6 cells. The increased level of P-STAT1 was blocked by the JAK inhibitor. Meanwhile, the level of overexpressed FL-MAVS was not affected by the JAK inhibitor (Fig. 3C). In line with the abrogation of P-STAT1, the anti-HEV capability of FL-MAVS was also diminished by the JAK inhibitor, as detected using an antibody against the HEV open reading frame 2 protein (HEV ORF2) (Fig. 3C). The reverse effect of the JAK inhibitor on HEV was confirmed in U87-p6-Luc cells (Supplementary Fig. 2A and B), a neural cell model used for studying HEV replication [14, 18]. Finally, the induction status of ISGs during combined treatment with FL-MAVS and JAK inhibitor was evaluated. We observed that the increased ISG induction by FL-MAVS was reversed by the JAK inhibitor in Huh7.5-p6 cells (Fig. 3D) and U87-p6-Luc cells (Supplementary Fig. S3C and D). Taken together, we discovered that overexpression of FL-MAVS inhibited HEV replication. Mechanistically, the anti-HEV effect of FL-MAVS is largely dependent on the activation of JAK-STAT signaling.

**Fig. 3:**
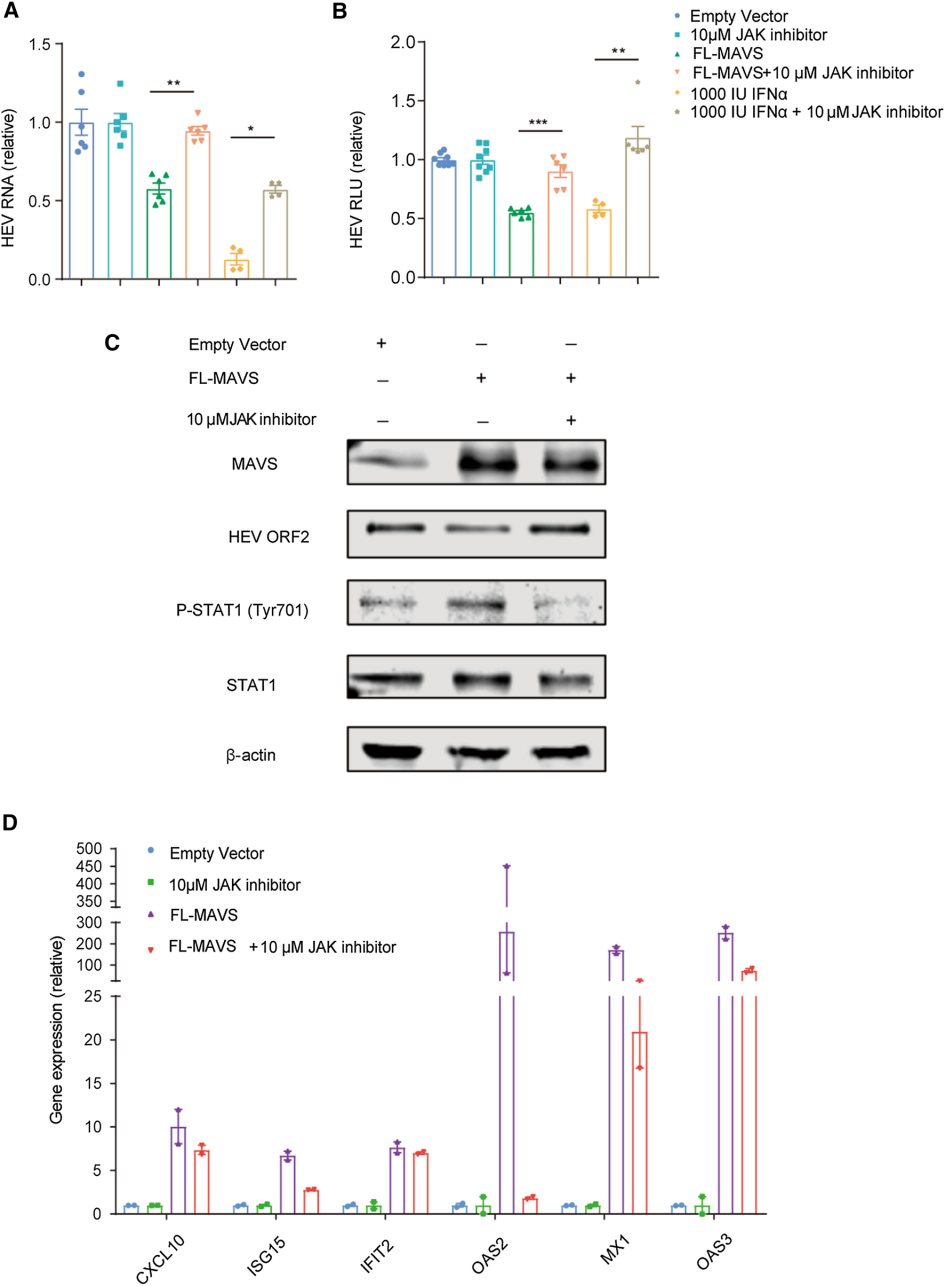
The antiviral effect of FL-MAVS against HEV is largely dependent on JAK-STAT activation. (A and B) An Huh7.5-p6 infectious model (n = 4-6) (A) or Huh7.5-p6-Luc replicon model (n = 4-8) (B) was transduced with FL-MAVS or empty vector or treated with 10 μM JAK inhibitor, 1000 IU IFNα, or 0.1% DMSO vehicle control. HEV RNA (48 h treatment) and luciferase expression (72 h treatment) was measured by quantitative RT-PCR. Data in the FL-MAVS group are presented relative to the empty vector group (set as 1). Data in the FL-MAVS group are presented relative to the empty vector group (set as 1). Data in the combination group of FL-MAVS with JAK inhibitor or IFNα are presented relative to JAK inhibitor- or IFNα-alone-treated group (set as 1). The data are the mean ± SEM (*, *P* < 0.05; **, *P* <0.01; ***, *P* < 0.001). (C) Immunoblot analysis of the indicated protein levels in Huh7-p6 cells transfected with empty vector (1 μg) or FL-MAVS vector (1 μg) or treated with JAK inhibitor for 48 h. MAVS was detected using an anti-MAVS antibody. (D) Quantitative RT-PCR analysis of ISG RNA in Huh7.5-p6 cells transduced with FL-MAVS or empty vector (n = 2) or treated with the indicated compounds for 48 h. Data in the FL-MAVS group are presented relative to the empty vector group (set as 1). Data in the combination group of FL-MAVS with JAK inhibitor are presented relative to the JAK inhibitor-alone-treated group (set as 1). The data are the mean ± SEM (*, *P* < 0.05; **, *P* <0.01; ***, *P* < 0.001).

MAVS serves as a vital adapter, linking mitochondria to the antiviral IFN response [19]. In turn, the MAVS-mediated IFN response is inhibited by various virus infections. For example, the Zika virus nonstructural proteins NS3 and NS2B3 have been shown to negatively regulate the IFN response by targeting MAVS [20]. The PB1-F2 protein of H7N9 influenza A virus can inhibit innate immunity by preventing MAVS aggregation [21]. Additionally, a nonstructural protein of Andes orthohantavirus (ANDV) was found to antagonize the IFN response by reducing MAVS ubiquitination, a posttranslational mechanism of protein modification mediating MAVS aggregation [22]. Most recently, a SARS-CoV-2 membrane glycoprotein has been found to negatively regulate innate immunity by impairing MAVS aggregation in HEK293 cells [23]. In the context of HEV infection, a previous study showed that HepG2 cells harboring an HEV replicon yielded MAVS polymer, which was not found in control cells, suggesting a potential role of MAVS aggregation in the inhibition of HEV replication [12]. Classically, the activation of JAK-STAT signaling requires the secretion of IFNs. The secreted IFNs bind to interferon receptors, leading to activation of the JAK-STAT pathway and a subsequent increase in expression of ISGs. This is thought to further enhance IFN production, which in turn leads to stronger activation of ISGs and restricts viral infection through positive feedback loops [24]. Our previous studies showed that RIG-I and MDA5 inhibited HEV through stimulation of ISGs production. However, these inhibitory effects do not require IFN secretion [9, 15]. These IFN-independent RLR effects might be explained by the observations that RIG-I restricts viral replication directly by inhibiting the binding of the viral polymerase to viral RNA [25] and that RIG-I and MDA5 can displace viral proteins bound to dsRNA [26]. Extended from our previous findings, we demonstrate here that overexpression of FL-MAVS also potently inhibits HEV replication by stimulating ISG expression. However, FL-MAVS could induce secretion of IFNs, and the anti-HEV activity of FL-MAVS was largely dependent on JAK-STAT activation. Of note, it seems that the JAK inhibitor exerts a wider range of inhibition of FL-MAVS-induced ISGs than IFN receptor inhibitors (B18R or 136R) (Fig. 3D and Supplementary Fig. 2E). This may explain why the JAK inhibitor reversed the anti-HEV effect of FL-MAVS, whereas IFN receptor inhibitors showed only a minor effect. In a previous study, Dixit et al. showed that peroxisomal MAVS does not induce IFN secretion or play a major role in IFN-independent ISG expression, whereas mitochondrial MAVS appears to be involved in IFN-dependent ISG production [7]. Based on these findings, we speculate that the overexpressed FL-MAVS may be localized in both mitochondria and peroxisomes in Huh7.5-p6 cells. Furthermore, the FL-MAVS in the different compartments may exert their anti-HEV effects through IFN-dependent or independent mechanisms. Dixit et al. also demonstrated that IFN regulatory factor 1 (IRF1) plays a key role in MAVS-dependent signaling in peroxisomes. In apparent agreement, our previous study revealed that IRF1 effectively inhibits HEV replication independently of IFN production [27]. More research is needed to determine whether IRF1 participates in the FL-MAVS-mediated anti-HEV activity. In summary, our present study provides further evidence of the vital role of the RIG-I/MDA5-MAVS signaling pathway in the inhibition of HEV replication. However, more information is required on the distinct mechanistic mode-of-action aspects of FL-MAVS in HEV replication. Moreover, the noncanonical antiviral mechanisms of this signaling pathway need further clarification, although the IFN-dependent mechanisms also need to be investigated further.

## Supplementary Information

Below is the link to the electronic supplementary material.

Supplementary Fig. 1 (A) Quantitative RT-PCR analysis of ISGs RNA in PLC-p6 cells transduced with FL-MAVS or empty vector for 48 h (n = 2-4). (B) Analysis of ISRE-related firefly luciferase activity in Huh7.5-ISRE-Luc cells transfected with empty vector (300 ng) or FL-MAVS vector (300 ng) for the indicated time periods (n = 2-3). (C) Quantitative RT-PCR analysis of IFN RNA in PLC-p6 cells transduced with FL-MAVS or empty vector (n = 4) for 48 h. PLC-p6 cells were transduced with FL-MAVS or empty vector for 72 h, and the cells were washed five times before the medium was refreshed. After another 72 h, the supernatant was collected as a conditioned medium. (D and E) Analysis of luciferase activity in Huh7.5-ISRE-Luc cells (n = 8) (D) or the HCV replicon model (n = 12) (E) treated with PLC-p6-derived conditioned medium for 72 h. (F) Analysis of luciferase activity in the HEV replicon model treated with Huh7.5-p6-derived conditioned medium for 48 h (n = 4). The data are the mean ± SEM (*, P < 0.05; **, P < 0.01; ***, P < 0.001), and the empty vector group served as a control (set as 1) (TIF 944 KB)

Supplementary Fig. 2 (A and B) Analysis of HEV-related luciferase activity in U87-p6-Luc cells transduced with FL-MAVS or empty vector (n = 2) or treated with a JAK inhibitor for 48 h (A) or 72 h (B). Data in the FL-MAVS group are presented relative to the empty vector group (set as 1). Data in the combination group of FL-MAVS with JAK inhibitor are presented relative to the JAK inhibitor-only-treated group (set as 1). (C and D) Quantitative RT-PCR analysis of ISGs RNA in U87-p6-Luc cells transduced with FL-MAVS or empty vector (n=6-8) or treated with the JAK inhibitor for 48 h. Data in the FL-MAVS group are presented relative to the empty vector group (set as 1). Data in the combination group of FL-MAVS with JAK inhibitor are presented relative to the JAK inhibitor-only-treated group (set as 1). (E) Quantitative RT-PCR analysis of ISG RNA in Huh7.5-p6 cells transduced with FL-MAVS or empty vector (n = 6-8) or treated with the indicated compounds for 48 h. Data in the FL-MAVS group are presented relative to the empty vector group (set as 1). Data in the combination group of FL-MAVS with 136R or B18R are presented relative to the 136R- or B18R-only-treated group (set as 1). The data are the mean ± SEM (*, P < 0.05; **, P <0.01; ***, P < 0.001) (TIF 889 KB)

Materials and methods In this study, the detailed Materials and methods are provided as a supplementary file (DOC 86 KB)
