# Supplementary figures and images for: Full-length MAVS, a mitochondrial antiviral-signaling protein, inhibits hepatitis E virus replication, requiring JAK-STAT signaling
Source: Arch Virol. 2022 Mar 24;167(5):1293–300. doi: 10.1007/s00705-022-05415-9 (PMC8942808; doi:10.1007/s00705-022-05415-9)

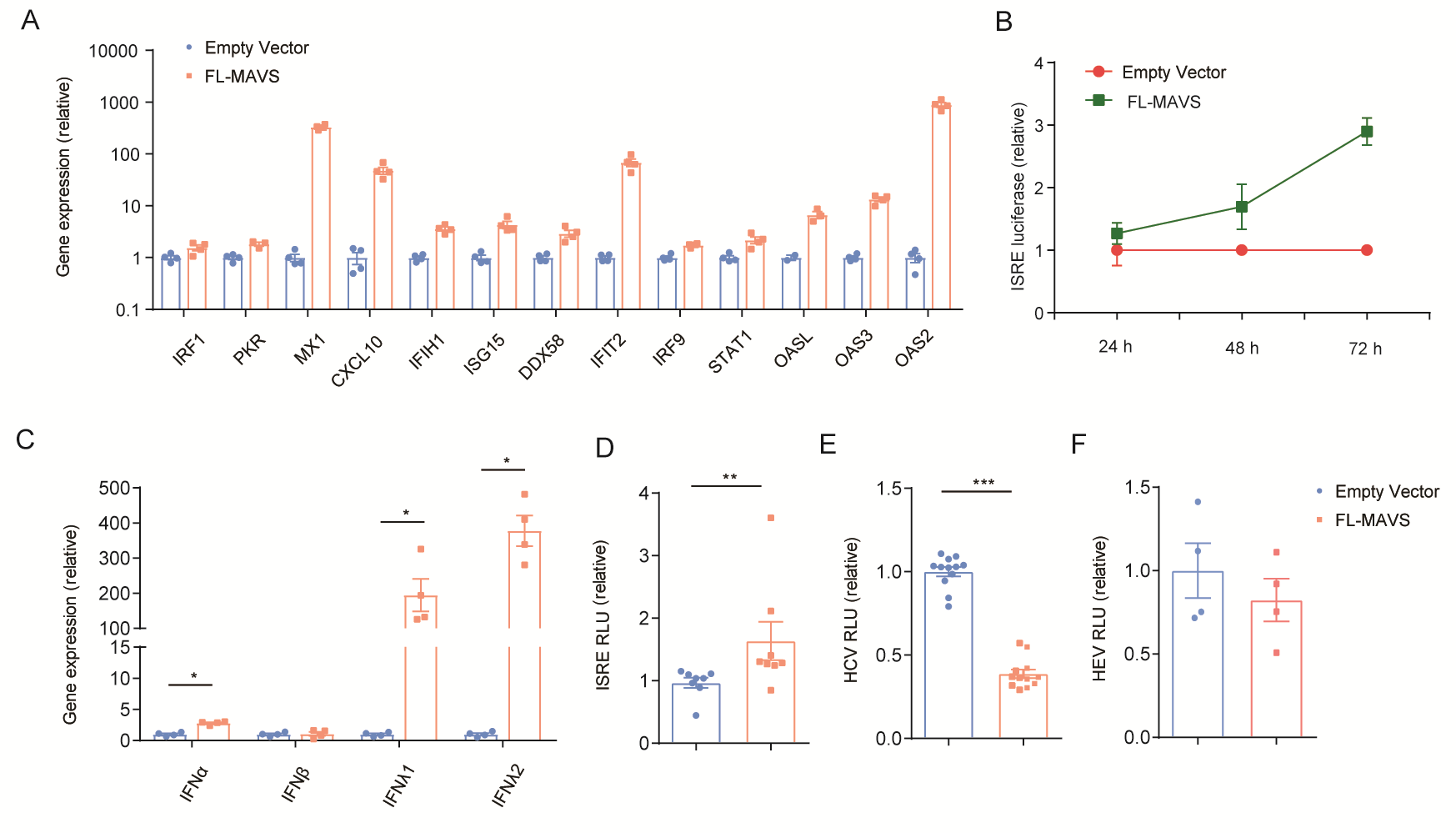

Supplement: Supplementary file 1 — Supplementary Fig. 1 (A) Quantitative RT-PCR analysis of ISGs RNA in PLC-p6 cells transduced with FL-MAVS or empty vector for 48 h (n = 2-4). (B) Analysis of ISRE-related firefly luciferase activity in Huh7.5-ISRE-Luc cells transfected with empty vector (300 ng) or FL-MAVS vector (300 ng) for the indicated time periods (n = 2-3). (C) Quantitative RT-PCR analysis of IFN RNA in PLC-p6 cells transduced with FL-MAVS or empty vector (n = 4) for 48 h. PLC-p6 cells were transduced with FL-MAVS or empty vector for 72 h, and the cells were washed five times before the medium was refreshed. After another 72 h, the supernatant was collected as a conditioned medium. (D and E) Analysis of luciferase activity in Huh7.5-ISRE-Luc cells (n = 8) (D) or the HCV replicon model (n = 12) (E) treated with PLC-p6-derived conditioned medium for 72 h. (F) Analysis of luciferase activity in the HEV replicon model treated with Huh7.5-p6-derived conditioned medium for 48 h (n = 4). The data are the mean ± SEM (*, P < 0.05; **, P < 0.01; ***, P < 0.001), and the empty vector group served as a control (set as 1) (TIF 944 KB) [file 705_2022_5415_MOESM1_ESM.tif]

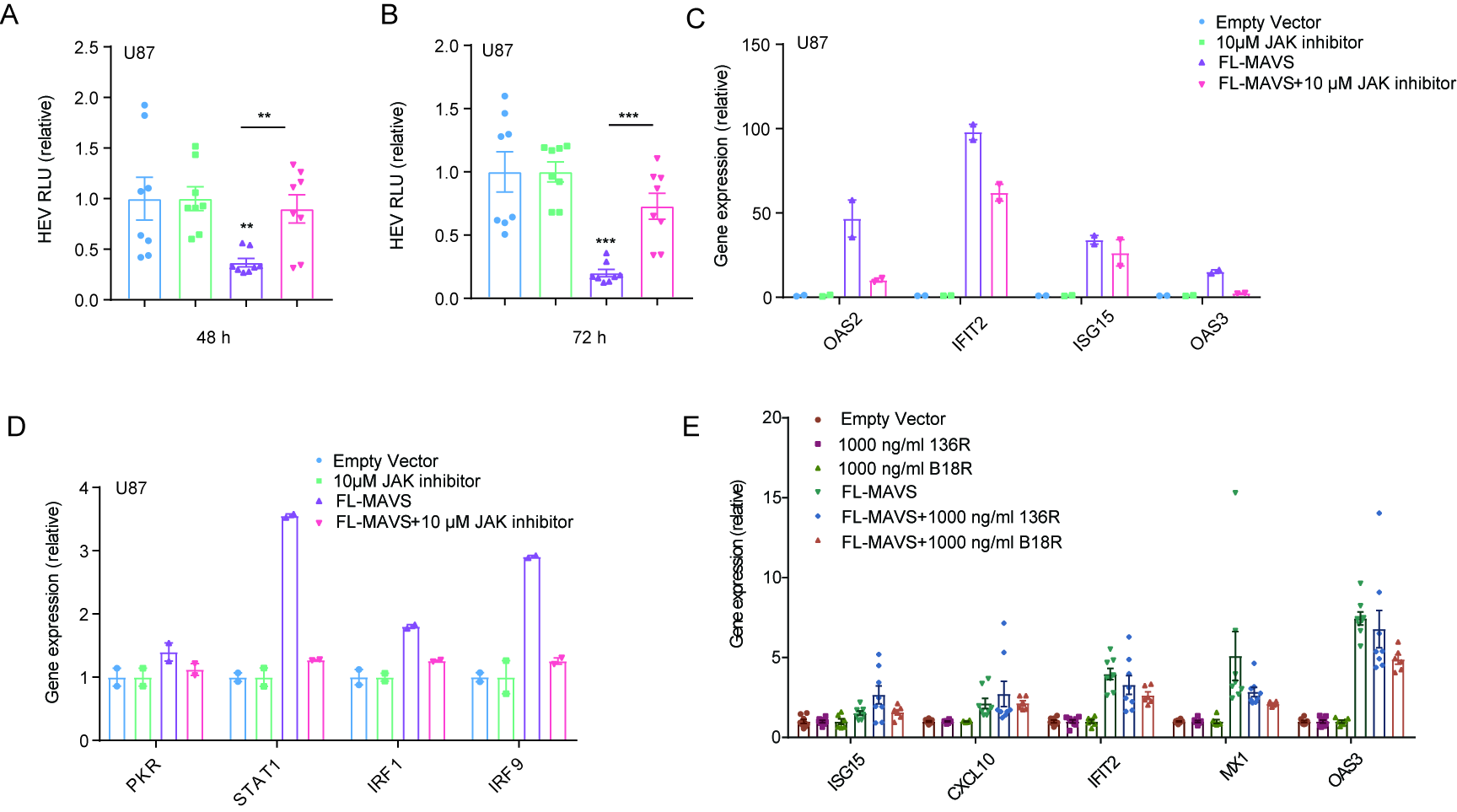

Supplement: Supplementary file 2 — Supplementary Fig. 2 (A and B) Analysis of HEV-related luciferase activity in U87-p6-Luc cells transduced with FL-MAVS or empty vector (n = 2) or treated with a JAK inhibitor for 48 h (A) or 72 h (B). Data in the FL-MAVS group are presented relative to the empty vector group (set as 1). Data in the combination group of FL-MAVS with JAK inhibitor are presented relative to the JAK inhibitor-only-treated group (set as 1). (C and D) Quantitative RT-PCR analysis of ISGs RNA in U87-p6-Luc cells transduced with FL-MAVS or empty vector (n=6-8) or treated with the JAK inhibitor for 48 h. Data in the FL-MAVS group are presented relative to the empty vector group (set as 1). Data in the combination group of FL-MAVS with JAK inhibitor are presented relative to the JAK inhibitor-only-treated group (set as 1). (E) Quantitative RT-PCR analysis of ISG RNA in Huh7.5-p6 cells transduced with FL-MAVS or empty vector (n = 6-8) or treated with the indicated compounds for 48 h. Data in the FL-MAVS group are presented relative to the empty vector group (set as 1). Data in the combination group of FL-MAVS with 136R or B18R are presented relative to the 136R- or B18R-only-treated group (set as 1). The data are the mean ± SEM (*, P < 0.05; **, P <0.01; ***, P < 0.001) (TIF 889 KB) [file 705_2022_5415_MOESM2_ESM.tif]
