# Supplementary material for: Full-length MAVS, a mitochondrial antiviral-signaling protein, inhibits hepatitis E virus replication, requiring JAK-STAT signaling
Source: Arch Virol. 2022 Mar 24;167(5):1293–300. doi: 10.1007/s00705-022-05415-9 (PMC8942808; doi:10.1007/s00705-022-05415-9)
Supplement: Supplementary file 3 — Materials and methods In this study, the detailed Materials and methods are provided as a supplementary file (DOC 86 KB) [file 705_2022_5415_MOESM3_ESM.doc]

**Material and methods**

**Reagents**

Human IFNα (Sigma-Aldrich) was dissolved in PBS. JAK inhibitor 1 (Santa Cruz Biotechnology, Santa Cruz, CA, USA) was dissolved in DMSO with a final concentration of 5 mg/ml. 136R (8976-BR-025) was obtained from Bio-techne. B18R (34-8185-81) was obtained from Invitrogen. 0.1% dimethyl sulfoxide (DMSO, Sigma, Zwijndrecht, the Netherlands) was used as vehicle control. FLAG antibody (mouse monoclonal, T510-2) was obtained from Signalway Antibody. P-STAT1 (Tyr701) (Rabbit mAb, 9167) and STAT1 (Rabbit mAb, 9172) antibodies were obtained from Cell Signaling Technology (Danvers, MA, USA). MAVS antibody (mouse monoclonal, sc-166583) and β-actin antibody (mouse monoclonal, sc-47778) were obtained from Santa Cruz Biotechnology (Santa Cruz, CA, USA). HEV ORF2 protein antibody (MAB8002) was obtained from EMD Millipore. Anti-mouse IRDye-conjugated secondary antibodies (Li-Cor Biosciences, Lincoln, NE, USA) were also used.

**Cell culture models**

Huh7.5, PLC human hepatoma cells, and U87 human glioblastoma cells were cultured in DMEM (Lonza Bio Whittaker, Verviers, Belgium) supplemented with 10% (v/v) fetal calf serum (FCS) (Hyclone, Logan, UT, USA), 100 IU/ml penicillin and 100 mg/ml streptomycin. The cells were cultured at 37 ℃ in a humidified atmosphere containing 5% CO2. A plasmid construct containing a subgenomic HEV sequence coupled with a Gaussia luciferase reporter gene (p6-Luc) or a construct containing the full-length HEV genome (p6) were used to generate genotype 3 HEV genomic RNA using the mMessage mMachine In Vitro RNA Transcription Kit (Thermo Fisher Scientific, Waltham, MA, USA) [1]. Huh7.5 and U87 cells were electroporated with p6-Luc subgenomic HEV RNA to generate genotype 3 HEV replicon models (Huh7.5-p6-Luc and U87-p6-Luc). Huh7.5 and PLC cells were electroporated with p6 HEV RNA to generate genotype 3 HEV infectious models (Huh7.5-p6 and PLC-p6). For genotype 1 HEV replicon model, Huh7.5 cells were electroporated with Sar 55/S17/Luc HEV RNA, and viral replication was detected by Gaussia luciferase activity (Genotype 1 HEV RLU). For the HCV replicon model, Huh7.5 cells electroporated with the sub-genomic HCV bicistronic replicon (I389/NS3–3V/LucUbiNeo-ET) were used. HCV replication was monitored by measuring firefly luciferase activity (HCV RLU) [2]. For the ISRE reporter model, Huh7.5 cells were transduced with a lentiviral transcriptional reporter system expressing the firefly luciferase gene driven by a promoter containing multiple ISRE promoter elements (SBI Systems Biosciences, Mountain View, CA, USA). Luciferase activity represents ISRE promoter activation [3]. The plasmids used to generate genotype 3 and genotype 1 HEV RNA were kind gifts from Dr. Suzanne U. Emerson (U.S. National Institutes of Health, National Institute of Allergy and Infectious Diseases). Prof. Ralf Bartenschlager and Dr. Volker Lohmann (University of Heidelberg, Germany) provided the HCV replicon cells. Prof. Dr. Charles M. Rice (The Rockefeller University) provided the ISRE lentiviral vector. Huh7.5 cells and PLC cells were electroporated using the Bio-Rad’s electroporation systems (240V, pulse length 0.5, number 1 and cuvette 4 mm) with HEV viral RNA to generate related cell models. Briefly, 1.5×10^6^ cells were collected and washed with 5 mL Opti-MEM (Thermo Fisher Scientific Life Sciences) three times. The cell pellet was resuspended with 100 µL Opti-MEM and mixed with 10 µg HEV RNA before being subjected to electroporation.

**Lentivirus production and transduction assays**

pCDH-EF-puro-CMV-MCS-based lentiviral vector targeting FL-MAVS, and non-targeted empty vector were a kind gift from Pinghui Feng (University of Southern California, Los Angeles, California, United States of America). Lentiviral pseudoparticles were generated in 293T cells as previously described [2]. Next, a supernatant that contains lentiviral pseudoparticles was ultracentrifuged (22000 rpm, 2 h) to concentrate lentiviral pseudoparticles (SW28 rotor; Beckman Coulter, Brea CA, USA). For transduction assays, cells were seeded into 96-well plates at a density of 1×10^4^ cells per well and transduced with concentrated lentiviral pseudoparticles at 37 °C. The FL-MAVS plasmid was also used for direct transfection to overexpress FL-MAVS before detecting the ISRE luciferase activity.

**Measurement of luciferase activity**

For the HEV RLU and Genotype 1 HEV RLU, the activity of secreted Gaussia luciferase in the cell culture medium was measured with the BioLux Luciferase Flex Assay Kit (New England Biolabs, Ipswich, MA, USA). For the ISRE RLU and HCV RLU, the activity of firefly luciferases was measured by adding luciferin potassium salt (100 mM; Sigma-Aldrich) to the cells and incubated for 10 min at 37°C. All luciferase activity was quantified with a LumiStar Optima Luminescence Counter (BMG LabTech, Offenburg, Germany).

**Real-time quantitative RT-PCR (Real-time qRT-PCR)**

RNA was isolated using the Machery-NucleoSpin RNA II kit (Bioke, Leiden, Netherlands). RNA concentration was quantified by a Nanodrop ND-1000 Spectrophotometer (Thermo, DE, USA). cDNA was prepared using Takara cDNA Synthesis Kit with random hexamer primers according to manufacturer’s instructions (Takara Bio, Inc., Shiga, Japan). Host gene expression and intracellular HEV level were quantified by SYBR-Green-based (Applied Biosystems® SYBR® Green PCR Master Mix, Thermo Fisher Scientific Life Sciences) real-time PCR. GAPDH was used as housekeeping genes and the expression levels of target genes were normalized to GAPDH by the 2^-ΔΔCT^ method. Primers sets used for this study were listed in Supplementary Table.

**Western blot assay**

Whole-cell lysates were heated at 95°C for 8 min, followed by loading onto a 15% SDS-polyacrylamide gel and separating by electrophoresis. After separation on a 12% SDS-PAGE gel, the proteins were transferred onto a PVDF membrane (Thermo Fisher Scientific). Subsequently, the membrane was blocked for 1 h at room temperature followed by incubation with anti-FLAG (1:2000) antibody overnight at 4°C. The membrane was washed 3 times followed by incubation for 1.5 h with a peroxidase-conjugated secondary antibody (1:10000). After the membrane was washed 3 times, the protein bands were detected with the Odyssey 3.0 Infrared Imaging System (Li-Cor, Lincoln, NE, USA).

**Transfection assay**

FuGENE® HD Transfection Reagent (Promega) was used for transfection assays. Cells were seeded at 80% confluence per well (using 96-well plates for Supplementary Figure 1B and 24-well plates for Figure 3C). After 24 h, the medium was removed and the cell layer was washed by Opti-MEM. The empty vector or FL-MAVS vector was transfected with FuGENE® HD Transfection Reagent in a total volume of 100 μL Opti-MEM according to the protocol. After 5 h, the medium was changed back to a normal medium.

**IFN production bioassay**

IFN production bioassay was conducted as a previous protocol [3]. In brief, Huh7.5-p6 were seeded into 6-well plates at a density of 1 × 10^5^ cells per well and transduced with FL-MAVS or empty vector at 37 °C. After 72 h, the cells were washed 5 times with PBS and the cell culture medium was refreshed. After another 72 h, the conditioned medium (supernatant) was subsequently collected and filtered through a 0.45 μm pore size membrane.

**Statistical analysis**

GraphPad Prism 5 software was used for data analysis using a Mann-Whitney U test. All results are presented as means ± SEM. Differences were significant at P<0.05.

**References:**

1. Qu C, Zhang S, Wang W, Li M, Wang Y, van der Heijde-Mulder M, Shokrollahi E, Hakim MS, Raat NJH, Peppelenbosch MP, Pan Q (2019) Mitochondrial electron transport chain complex III sustains hepatitis E virus replication and represents an antiviral target. FASEB journal : official publication of the Federation of American Societies for Experimental Biology 33:1008-1019

2. Xu L, Wang W, Li Y, Zhou X, Yin Y, Wang Y, de Man RA, van der Laan LJW, Huang F, Kamar N, Peppelenbosch MP, Pan Q (2017) RIG-I is a key antiviral interferon-stimulated gene against hepatitis E virus regardless of interferon production. Hepatology (Baltimore, Md) 65:1823-1839

3. Li Y, Yu P, Qu C, Li P, Li Y, Ma Z, Wang W, de Man RA, Peppelenbosch MP, Pan Q (2020) MDA5 against enteric viruses through induction of interferon-like response partially via the JAK-STAT cascade. Antiviral research 176:104743

**Supplementary Table. Primer sequences**

| Genes | Sequences (5’ to 3’) |
| --- | --- |
| HEV-F | ATCGGCCAGAAGTTGGTTTTTAC |
| HEV-R | CCGTGGCTATAACTGTGGTCT |
| GAPDH-F | GTCTCCTCTGACTTCAACAGCG |
| GAPDH-R | ACCACCCTGTTGCTGTAGCCAA |
| MAVS-F | TACCAGAGCTACCAGCCTC |
| MAVS-R | GGGGTAACTTGGCTCCTTCT |
| IRF1-F | GAGGAGGTGAAAGACCAGAGCA |
| IRF1-R | TAGCATCTCGGCTGGACTTCGA |
| PKR-F | GAAGTGGACCTCTACGCTTTGG |
| PKR-R | TGATGCCATCCCGTAGGTCTGT |
| MX1-F | GGCTGTTTACCAGACTCCGACA |
| MX1-R | CACAAAGCCTGGCAGCTCTCTA |
| CXCL10-F | GGTGAGAAGAGATGTCTGAATCC |
| CXCL10-R | GTCCATCCTTGGAAGCACTGCA |
| IFIH1-F | GCTGAAGTAGGAGTCAAAGCCC |
| IFIH1-R | CCACTGTGGTAGCGATAAGCAG |
| ISG15-F | CTCTGAGCATCCTGGTGAGGAA |
| ISG15-R | AAGGTCAGCCAGAACAGGTCGT |
| DDX58-F | CACCTCAGTTGCTGATGAAGGC |
| DDX58-R | CACCTCAGTTGCTGATGAAGGC |
| IFIT2-F | GGAGCAGATTCTGAGGCTTTGC |
| IFIT2-R | GGATGAGGCTTCCAGACTCCAA |
| IRF9-F | CCACCGAAGTTCCAGGTAACAC |
| IRF9-R | AGTCTGCTCCAGCAAGTATCGG |
| STAT1-F | ATGGCAGTCTGGCGGCTGAATT |
| STAT1-R | CCAAACCAGGCTGGCACAATTG |
| OASL-F | GTGCCTGAAACAGGACTGTTGC |
| OASL-R | CCTCTGCTCCACTGTCAAGTGG |
| OAS3-F | CCTGATTCTGCTGGTGAAGCAC |
| OAS3-R | TCCCAGGCAAAGATGGTGAGGA |
| OAS2-F | GCTTCCGACAATCAACAGCCAAG |
| OAS2-R | CTTGACGATTTTGTGCCGCTCG |
